# Constitutive Damage Model for Rubber Fiber-Reinforced Expansive Soil under Freeze–Thaw Cycles

**DOI:** 10.3390/ma17204979

**Published:** 2024-10-11

**Authors:** Rongchang Wang, Zhongnian Yang, Xianzhang Ling, Wei Shi, Zhenxing Sun, Xipeng Qin

**Affiliations:** 1 School of Civil Engineering, Qingdao University of Technology, Qingdao 266520, China; wrcoan@163.com (R.W.); lingxianzhang@qut.edu.cn (X.L.); shiwei123@qut.edu.cn (W.S.); sun.zhenxing@outlook.com (Z.S.); 2 School of Civil Engineering, Harbin Institute of Technology, Harbin 150006, China; xipeng_qin@163.com

**Keywords:** freeze–thaw cycle, rubber fiber, expansive soil, elastic modulus, damage constitutive model

## Abstract

To elucidate the degradation mechanism of expansive soil–rubber fiber (ESR) under freeze–thaw cycles, freeze–thaw cycle tests and consolidated undrained tests were conducted on the saturated ESR. The study quantified the elastic modulus and damage variables of ESR under different numbers of freeze–thaw cycles and confining pressure, and proposed a damage constitutive model for ESR. The primary findings indicate that: (1) The effective stress paths of ESR exhibit similarity across different numbers of freeze–thaw cycles, the critical stress ratio slightly decreased by 8.8%, while the normalized elastic modulus experienced a significant reduction, dropping to 42.1%. (2) When considering the damage threshold, the shear process of ESR can be divided into three stages: weak damage, damage development, and failure. As strain increases, the microdefects of ESR gradually develop, penetrating macroscopic cracks and converging to form the main rupture surface. Eventually, the damage value reaches 1. (3) Due to the effect of freeze–thaw cycles, initial damage exists for ESR, which is positively correlated with the number of freeze–thaw cycles. The rubber fibers act as tensile elements, and the ESR damage evolution curves intersect one after another, showing obvious plastic characteristics in the late stage of shear. (4) Confining pressure plays a role in limiting the development of ESR microcracks. The damage deterioration of ESR decreases with an increase in confining pressure, leading to an increase in ESR strength. (5) Through a comparison of the test curve and the theoretical curve, this study validates the rationality of the damage constitutive model of ESR under established freeze–thaw cycles. Furthermore, it accurately describes the nonlinear impact of freeze–thaw cycles and confining pressure on the ESR total damage.

## 1. Introduction

Expansive soil is rich in hydrophilic minerals such as montmorillonite and illite, demonstrating significant expansion–contraction potential, particularly in volume changes induced by environmental factors (freeze–thaw cycles and dry–wet cycles) [1]. The typical characteristic of volume changes involves expansion through water absorption and contraction through water loss [2], leading to cracking and failure of foundations [3], roadbeds [4], dams [5], and channels [6]. Expansive soil causes more damage to buildings than any other natural disaster, including earthquakes and floods. It has been estimated that worldwide losses due to expansive soil reach billions of dollars, including GBP 150 million in the UK, USD 1 billion in the USA, and USD 4 million in South Africa [7].

In cold regions, the most crucial factor determining the engineering behavior of expansive soil is the freeze–thaw cycle. The freeze–thaw cycle significantly impacts the microstructure and mechanical properties of soil, including pore distribution, porosity [8,9,10], and strength [11,12,13,14], among others. Specifically, due to the action of freeze–thaw cycles, the microstructure of the aggregate transitions from dense to porous, and the pores become interconnected [15]; soil particles break, leading to an expansion of soil porosity and the formation of cracks between aggregated particles [16]; strength, stiffness, and viscosity gradually weaken to the critical values of soil stability [17]. Therefore, the issue of soil improvement, particularly in regions with high freeze–thaw cycles, has become a focal point in the geotechnical academic community.

As one of the largest sources of solid waste, the growing amount of scrap tires has led to serious disposal challenges. It is estimated that there are 1 billion waste tires worldwide each year, and this number will continue to increase to 1.2 billion by 2030 [18], indicating a significant surplus of waste tires available for recycling and beneficial reuse. Previous studies have demonstrated that waste-tire products, such as rubber powder, rubber granules, rubber aggregates, and rubber fibers, are utilized to enhance expansive soil foundations, thereby contributing to resource conservation and environmental protection [19,20,21,22]. Similar to fiber-reinforced soil, rubber is randomly distributed in the soil, optimizing rubber content and shape to mitigate swelling potential [23,24,25], improve ductility [26,27], increase shear strength [28,29,30,31], and enhance the damping ratio [32,33,34]. Compared to composite materials such as synthetic fibers and geotextiles, rubber has significant advantages in reducing materials costs and minimizing environmental pollution [35]. Furthermore, waste rubber also addresses challenges faced during construction in geotechnical engineering.

Damage mechanics (DM) is a relatively new field of research focused on studying the mechanical response and reliability of materials weakened by numerous randomly distributed microcracks of irregular shape and orientation. Kachanov [36] first proposed the concept of damage mechanics in his study of creep fracture, assuming that the stiffness and damage caused by microcracks could be calculated from macroscopic damage parameters. Lu et al. [37] established an elastoplastic constitutive model of unsaturated undisturbed expansive soil that can reflect crack development based on unsaturated soil mechanics and damage mechanics. Yin et al. [38] developed a fractal derivative viscoelastic–plastic creep model considering the effect of damage and evaluated the creep damage mechanism through a series of triaxial consolidation drainage creep tests, where the sample exhibited attenuation creep at low stress, otherwise, it exhibited non-attenuation creep. De et al. [39] proposed an advanced numerical model based on the cohesive zone method and dynamic mesh technology for simulating crack propagation in quasi-brittle materials. Freeze–thaw damage evaluation indexes include porosity [40,41], longitudinal wave velocity [42,43], and elastic modulus [44,45,46]. Research has shown that rubber can mitigate the detrimental effects of freeze–thaw cycles on the mechanical properties of expansive soil [47,48,49]. Yang et al. [48] found that the shear stress of expansive soil–rubber fiber (ESR) under high confining pressure is slightly higher compared to ordinary expansive soil, and the damping ratio of ESR changes little under the influence of freeze–thaw cycles. Zhang et al. [49] investigated the crack evolution of ESR under freeze–thaw conditions and found that the addition of 5% rubber powder can effectively reduce crack evolution and surface crack rates. However, the aforementioned studies did not explicitly clarify the damage deterioration effects of freeze–thaw cycles on ESR, and the damage mechanism and evolution patterns have yet to be well described, indicating the need for further research.

Based on the above, the study aimed to explore the damage mechanism and evolution patterns of ESR under the coupled effects of freeze–thaw cycles and loading. The research quantifies the impact of freeze–thaw cycles on the stress ratio, normalized elastic modulus, and damage variable of ESR. In addition, a constitutive model for ESR damage has been established, taking into account the coupled effects of confining pressure and freeze–thaw cycles. The findings of this study offer a theoretical reference for the geological environment and geotechnical engineering in seasonal frozen soil areas.

## 2. Materials and Methods

**Figure 1:**
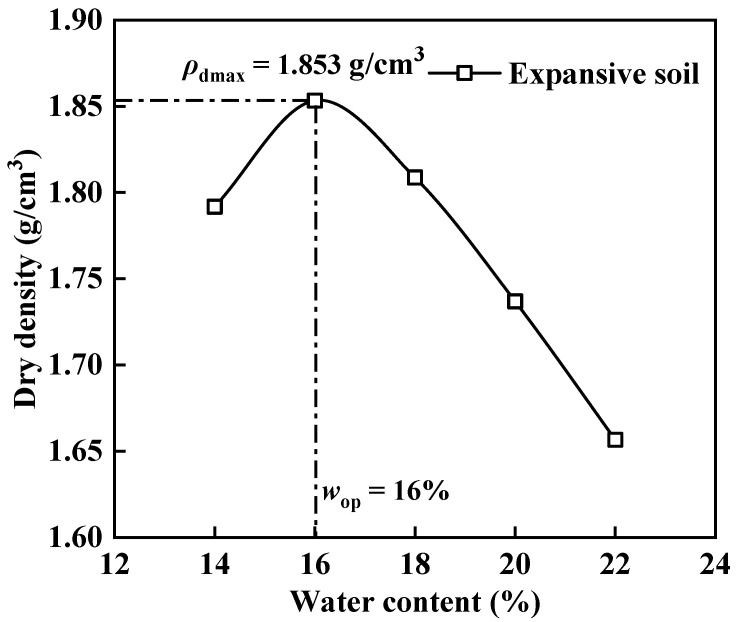
Compaction curve of expansive soil.

The expansive soil utilized in this study was remodeled expansive soil sourced from Henan province, China, and underwent geotechnical testing. The test results are detailed in Table 1. According to the classification of expansive soil by Habibbeygi and Nikraz [50], the soil falls under the category of highly plastic clay. The maximum dry density and optimum moisture content of the expansive soil are illustrated in Figure 1. Subsequently, the expansive soil was dried, crushed through a 1 mm sieve, and processed into soil powder for later use.

As a composite material, rubber possesses high elasticity, durability, and frictional resistance. The rubber fibers employed in the test were obtained from a tire treatment plant in Deyang, Sichuan, China. These rubber fibers underwent sieving to eliminate finer particles and larger debris, resulting in the selection of short fibers measuring approximately 15 mm in length and 1 mm in diameter. According to the fiber tensile test, the tensile strength of the rubber fibers is approximately 21.1 MPa.

Akbarimehr et al. [51] discovered that incorporating 10% rubber could optimize the damping ratio of clay. Consequently, in this study, rubber fiber contents of 0%, 5%, 10%, 15%, and 20% were mixed with expansive soil. The samples in this study were prepared at the optimum moisture content and then subjected to testing after saturation. The total mass was controlled and the samples were prepared using the stratified compaction method, which was used to improve the random uniform distribution of fibers in the samples. The soil was prepared at an optimal moisture content, following which the rubber fibers were evenly mixed with the expansive soil. The resulting mixture of fiber and expansive soil was then divided into five compacted layers to form the sample, ensuring the random and uniform distribution of rubber fibers within a sample measuring 39.1 mm in diameter and 80 mm in height. During the compaction process, the layers were scraped to promote a strong bond between adjacent layers of the mixture. The prepared sample underwent saturation using the pumping saturation method, followed by vacuuming in a vacuum saturation cylinder for 2 h, and subsequent immersion in water for 48 h. In this study, “*f*” is defined as the ratio of rubber to the total mass of rubber and soil, as depicted in Equation (1).

(1) $$f=\frac{m_{R}}{m_{s}+m_{\omega }+m_{R}}\times 100\%$$

**Figure 2:**
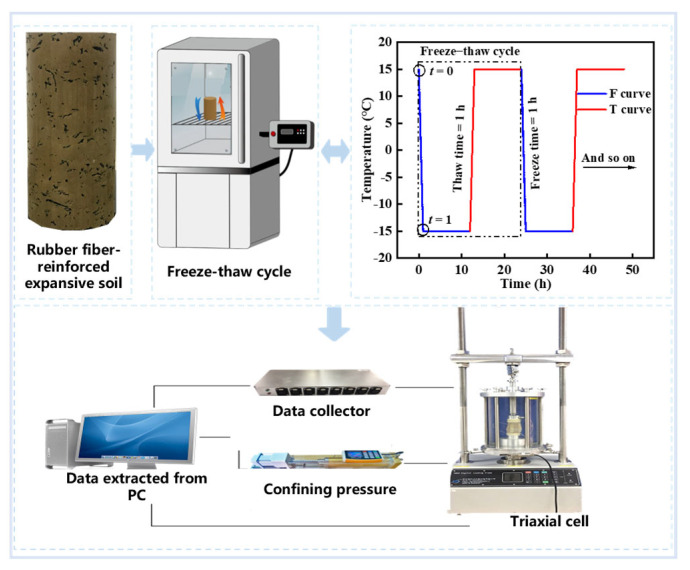
Flowchart of freeze–thaw cycle and consolidated undrained test.

According to the results of the triaxial tests conducted at normal temperatures [52], it was observed that the addition of rubber fiber could enhance the cohesion of expansive soil. The optimum rubber fiber content was determined to be 10%. Subsequently, freeze–thaw cycle and consolidation undrained tests were exclusively conducted out on expansive soil reinforced with 10% rubber fiber. The specific test program is outlined in Table 2. At −15 °C, the brittleness of the material can be observed under low temperature, while 15 °C reflects the recovery characteristics of the material at room temperature. This temperature difference helps to comprehensively evaluate the material’s performance at different temperatures, providing a reliable basis for practical applications. To mitigate volume changes resulting from frost heaving, the saturated samples were placed in a triaxial saturator (manufactured by Suzhou Yuchuang Fluid Technology Co., Ltd., Suzhou, China) [48], frozen in an TMS9015-800 constant temperature and humidity equipment (manufactured by Zhejiang Tomos Technology Co., Ltd., Ningbo, China) at −15 °C for 12 h, and then thawed before being left in the incubator at 15 °C for 12 h, constituting one freeze–thaw cycle as illustrated in Figure 2. Adjustable temperature-freezing chambers and temperature-controlled water baths were used to set the temperature range for freeze–thaw cycles, and multiple temperature sensors were installed inside the samples to monitor temperature changes at various positions in real-time.

Following the freeze–thaw cycles, the samples were subjected to a consolidated undrained triaxial test (CU). The testing apparatus employed was a TSZ automatic triaxial instrument (manufactured by Road Instrument Branch of Nanjing Soil Instrument Factory CO., LTD, Nanjing, China) equipped with a computer control system and automatic data acquisition system. Pore water pressure inside the sample was measured using a pressure transducer, while the load and strain were measured using a load cell and a linear variable differential transducer (LVDT). For slopes and subgrade projects involving expansive soil, only the soil depth below 10 m needs to be considered, where the confining pressure is relatively low. Therefore, consolidated undrained tests were conducted at confining pressures of 100, 200, and 300 kPa. When the pore water pressure dissipated to zero, consolidation was considered complete. Subsequently, an undrained shear test was conducted under displacement control at a rate of 0.08 mm/min. The specific flowchart of the freeze–thaw cycle and consolidated undrained test is shown in Figure 2.

The rubber fiber-reinforced expansive soil backfill was prepared in a rotating drum mixer, by mixing air-dried expansive soil, water (16% moisture content), and rubber fibers. Layers of this mixture were spread over the residual soil in consecutive sublayers, each 150 mm of maximum thickness, using a vibratory plate to reach a dry unit weight of 16.3 kN/m^3^ at 16% moisture content. Multiple samples were taken through equidistant sampling to check whether the rubber fibers were evenly distributed.

## 3. Damage Constitutive Model for ESR

Based on the strain equivalence hypothesis proposed by Lemaitre [53], effective stress is the primary factor affecting soil strain. In the event of material damage, the constitutive relation of the material requires an adjustment of stress to align with the effective stress, thereby establishing the foundational relationship of the ESR damage constitutive model:

(2) $$\sigma_{\mathrm{ij}}=\sigma^{*}\left(1-D\right)=E_{\mathrm{ijkl}}\varepsilon_{\mathrm{kl}}\left(1-D\right)$$

where *E*_ijkl_ is the tensor component of stiffness of the material; *σ*_ij_ is the tensor component of stress; σ* is the tensor component of effective stress; *ε*_kl_ is the tensor component of strain; and *D* is the damage variable.

From Equation (2), it is apparent that the establishment of the damage constitutive relation of ESR hinges on setting the damage variable *D*. In soil mechanics, the soil is often treated as a continuum with strong integrity and structure due to particle interlocking. Material damage occurs continuously during loading, leading to the following assumptions:

(1) The ESR unit contains microstructural damage (such as cracks, voids, etc.) but at the macro level, it is considered an isotropic, homogeneous, and continuous material.

(2) The ESR microelement can be viewed as linearly elastic before experiencing damage under loading, with minimal occurrence of new cracks or breakage within the samples. The stress–strain relationship adheres to Hooke’s law, allowing the initial tangent modulus to be replaced by the elastic modulus of the undamaged material.

(3) Failure strength in ESR is attributed to damage at the microstructural level, where the ratio of damaged area to total cross-sectional area is termed the microstructural damage variable. From a macro perspective, this can be defined in terms of the ratio of the number of damaged cells *N_f_* to the total number of cells *N*, as follows:

(3) $$D=\frac{N_{f}}{N}$$

Previous studies [54] have demonstrated that the Weibull distribution is better suited to describe the strength distribution pattern of clay under freeze–thaw cycles. By introducing the microelement strength as a random variable and integrating it with the Weibull distribution function, it can be obtained:

(4) $$\left\{\begin{array}{l} P\left(F^{*}\right)=\frac{\alpha }{F_{0}}{\left(\frac{F^{*}}{F_{0}}\right)}^{m-1}\exp\left[-{\left(\frac{F^{*}}{F_{0}}\right)}^{m}\right]F^{*}\geq 0\\ 0\;\;\;\;\;\;\;\;\;\;\;\;\;F^{*}\leq 0 \end{array}\right.$$

This gives the damage variable *D* as:

(5) $$D=\frac{N_{f}}{N}=\int_{-\infty }^{F}P\left(x\right)\mathrm{dx}=1-\exp\left[-{\left(\frac{F^{*}}{F_{0}}\right)}^{m}\right]$$

where *m* and *F*_0_ are the Weibull distribution parameters.

The microelement strength of ESR is determined using the Druck–Prager damage criterion, and the fundamental form of microelement damage of ESR is selected as follows:

(6) $$F^{*}=\alpha I_{1}^{*}+\sqrt{J_{2}^{*}}$$

(7) $$I_{1}^{*}=\sigma_{1}^{*}+\sigma_{2}^{*}+\sigma_{3}^{*}$$

(8) $$J_{2}^{*}=\frac{1}{6}\left[{\left(\sigma_{1}^{*}-\sigma_{2}^{*}\right)}^{2}+{\left(\sigma_{2}^{*}-\sigma_{3}^{*}\right)}^{2}+{\left(\sigma_{1}^{*}-\sigma_{3}^{*}\right)}^{2}\right]$$

Bringing Equation (6) into Equation (5), the damage evolution equation is as follows:

(9) $$D=1-\exp\left[-{\left(\frac{\alpha I_{1}^{*}+\sqrt{J_{2}^{*}}}{F_{0}}\right)}^{m}\right]$$

Associative Equations (2) and (9) can be obtained:

(10) $$\frac{1}{1-D}=\exp\left[{\left(\frac{\alpha I_{1}+\sqrt{J_{2}}}{F_{0}\left(1-D\right)}\right)}^{m}\right]$$

(11) $$I_{1}=\sigma_{1}+\sigma_{2}+\sigma_{3}$$

(12) $$J_{2}=\frac{1}{6}\left[{\left(\sigma_{1}-\sigma_{2}\right)}^{2}+{\left(\sigma_{2}-\sigma_{3}\right)}^{2}+{\left(\sigma_{1}-\sigma_{3}\right)}^{2}\right]$$

By $F=aI_{1}+\sqrt{J_{2}}$, it can be written in general form as:

(13) $$\frac{1}{1-D}=\exp\left[{\left(\frac{F}{F_{0}\left(1-D\right)}\right)}^{m}\right]$$

When *D* = 0, no damage occurs to the soil, and *F* = 0 is a prerequisite for the above equation to be valid. According to the Druck–Prager damage criterion, *a* > 0, thus, the prerequisite for the establishment is:

(14) $$\sigma_{1}=\sigma_{2}=\sigma_{3}=0$$

Equation (14) suggests that damage occurs when the soil is subjected to even a small external load. Upon analyzing the stress–strain curve of the sample, it becomes evident that the stress–strain relationship initially follows a linear pattern during the early stage of axial stress loading. However, at a certain point, the slope of the curve gradually decreases with the increase in stress, indicating a nonlinear relationship. This observation highlights a specific point in the stress–strain curve, beyond which the sample undergoes damage. The stress value corresponding to this point is considered the stress damage threshold, rendering Equation (14) unreasonable.

When the stress remains below the stress damage threshold, the stress–strain relationship maintains a linear progression, suggesting an absence of damage within the sample. However, once the stress surpasses the damage threshold, damage initiates within the soil and continues to intensify throughout the loading process.

Combining Equation (2) with Equation (10), we can express the stress–strain relationship of the soil under axisymmetric stress as follows:

(15) $$\sigma =E\varepsilon_{1}\exp\left[-{\left(\frac{F^{*}}{F_{0}}\right)}^{m}\right]+2\mu \sigma_{3}$$

Hence, the constitutive relationship of ESR under loading can be expressed as follows:

(16) $$\sigma =E\varepsilon_{1}\exp\left[-{\left(\frac{\alpha I_{1}+\sqrt{J_{2}}}{F_{0}}\right)}^{m}\right]+2\mu \sigma_{3}$$

where *E* is the elastic modulus; *ε*_1_ is the axial strain; *μ* is the Poisson’s ratio; and *σ*_3_ is the confining pressure.

The damage to ESR is a result of the combined effects of loading and freeze–thaw cycles, and the freeze–thaw damage variable can be described on a macroscopic scale. By introducing the elastic modulus as a physical quantity, the damage variable, which quantifies the number of freeze–thaw cycles, can be expressed as:

(17) $$D_{n}=1-\frac{E_{n}}{E_{0}}$$

where *E*_0_ is the elastic modulus of the soil without freeze–thaw cycles, and *E*_n_ is the elastic modulus of the soil after undergoing *n* freeze–thaw cycles. Preliminarily calculate the elastic modulus based on experimental data and utilize the least squares method for parameter optimization.

Taking into account the combined impact of freeze–thaw and load, the constitutive damage relationship of the ESR can be expressed as follows:

(18) $$\sigma =E\varepsilon \left(1-D_{m}\right)$$

(19) $$D_{m}\approx D+D_{n}-DD_{n}$$

where *D_m_* is the damage variable under the combined influence of load and freeze–thaw; *D* is the damage variable under load; and *D_n_* is the damage variable under freeze–thaw.

(20) $$D_{m}=1-\frac{E_{n}}{E_{0}}\exp\left[-{\left(\frac{\alpha I_{1}+\sqrt{J_{2}}}{F_{0}}\right)}^{m}\right]$$

The damage constitutive equation of ESR under the combined influence of freeze–thaw and load is as follows:

(21) $$\sigma_{1}=E_{n}\varepsilon_{1}\exp\left[-{\left(\frac{\alpha I_{1}+\sqrt{J_{2}}}{F_{0}}\right)}^{m}\right]+2\mu \sigma_{3}$$

Under the application of triaxial external force, a threshold point exists in the ESR damage process. Therefore, in this study, the strain corresponding to the threshold point is considered as the strain threshold *ɛ*_1*D*_. It has been observed that the relationship between *I*_1_ and $\sqrt{J_{2}}$ is nearly linear, thus defines $\sqrt{J_{2}=sI_{1}+t}$, the linear relationship is obtained as depicted in Table 3.

According to Table 3, the relationship between *s*, *t*, and the rubber fiber content *f* closely follows a power function, as shown below:

(22) $$s=0.0277\exp\left(1.9383f\right)$$

(23) $$t=1.0165f^{-0.8436}$$

The damage condition for ESR is:

(24) $$G=\sqrt{J_{2}}-sI_{1}-t$$

Associative Equations (22)–(24) give the damage condition for ESR as:

(25) $$G=\sqrt{J_{2}}-0.0277\exp\left(1.9383f\right)I_{1}-1.0165f^{-0.8436}$$

Considering the damage threshold, the evolution equation for the damage variable is as follows:

(26) $$D=\left\{\begin{array}{l} 0\;\;\;\;\;\;\;\;\;\;\;\;\;G<0\\ 1-\exp\left[-{\left(\frac{\alpha I_{1}^{*}+\sqrt{J_{2}^{*}}-F_{G}^{*}}{F_{0}}\right)}^{m}\right]\;G\geq 0 \end{array}\right.$$

where $F_{G}^{*}$ is the microelement strength of ESR corresponding to the damage threshold.

From Equations (2), (6), (21), (25) and (26), the constitutive equations for damage can be derived for ESR under freeze–thaw cycles, taking into account the damage threshold.

(27) $$\varepsilon_{1}=\left\{\begin{array}{l} \frac{\sigma_{1}-2\mu \sigma_{3}}{E_{n}}\;\;\;\;\;\;\;\;\;\;\;\;\;\;\;\;\;\;\;\;\;\;G<0\\ \frac{\sigma_{1}-2\mu \sigma_{3}}{E_{n}}\exp\left[{\left(\frac{\left(\begin{array}{l} \alpha I_{1}+\sqrt{J_{2}}-\left(\alpha +0.0277\exp\left(1.9383f\right)\right)\\ I_{1G}-1.0165f^{-0.8436}P_{a} \end{array}\right)E_{n}\varepsilon_{1}}{F_{0}\left(\sigma_{1}-2\mu \sigma_{3}\right)}\right)}^{m}\right]G\geq 0 \end{array}\right.$$

Simplified:

(28) $$\frac{\sigma_{1}-2\mu \sigma_{3}}{E_{n}\varepsilon_{1}}=\left\{\exp\left[-{\left(\frac{\left(\begin{array}{l} \alpha I_{1}+\sqrt{J_{2}}-\left(\alpha +0.0277\exp\left(1.9383f\right)\right)I_{1G}\\ -1.0165f^{-0.8436}P_{a} \end{array}\right)E_{n}\varepsilon_{1}}{F_{0}\left(\sigma_{1}-2\mu \sigma_{3}\right)}\right)}^{m}\right]\right.$$

Taking the logarithm of both sides of Equation (28), we obtain:

(29) $$\ln a+m\ln b=\ln\left[\frac{\sigma_{1}-2\mu \sigma_{3}}{E_{n}\varepsilon_{1}}\right]$$

(30) $$a={\left(\frac{1}{F_{0}}\right)}^{m}$$

(31) $$b=\frac{\left(\begin{array}{l} \alpha I_{1}+\sqrt{J_{2}}-\left(\alpha +0.0277\exp\left(1.9383f\right)\right)I_{1G}\\ -1.0165f^{-0.8436}P_{a} \end{array}\right)E_{n}\varepsilon_{1}}{\left(\sigma_{1}-2\mu \sigma_{3}\right)}$$

Defining $Y=\ln\left(-\frac{\sigma_{1}-2\mu \sigma_{1}}{E_{n}\varepsilon_{1}}\right)$; X=lnb; c=lna; we can transform Equation (31) into:

(32) Y=mX+c

(33) $$F_{0}=\exp\left(-\frac{c}{m}\right)$$

(34) $$\alpha =\frac{\sin\varphi }{\sqrt{3}\sqrt{\left(3+\sin^{2}\varphi \right)}}$$

(35) $$k=\frac{\sqrt{3}c\cos\varphi }{\sqrt{3+\sin^{2}\varphi }}$$

The constructed damage model requires the determination of six parameters: elastic modulus, internal friction angle, soil element strength *F*, damage variable, *m*, and *F*_0_. These parameters can be determined based on the results of the triaxial tests. From the stress–strain relationship curves of the ESR triaxial tests under different freeze–thaw cycle counts, the slope of the linear segment is taken as the elastic modulus. The known data are then substituted into Equation (6) to obtain the *F* value. The *F* value obtained from the previous step is substituted into Equation (32), and a fitting method is used to derive the parameters *m* and *c*. From the *c* value, the *F*_0_ value can be calculated. *α* and *k* are the constants related to the angle of internal friction *φ* and cohesion *c* in the geotechnical material.

## 4. Results

### 4.1. Analysis of Mechanical Properties of ESR

#### 4.1.1. Effect of Freeze–Thaw Cycles on Critical States

**Figure 3:**
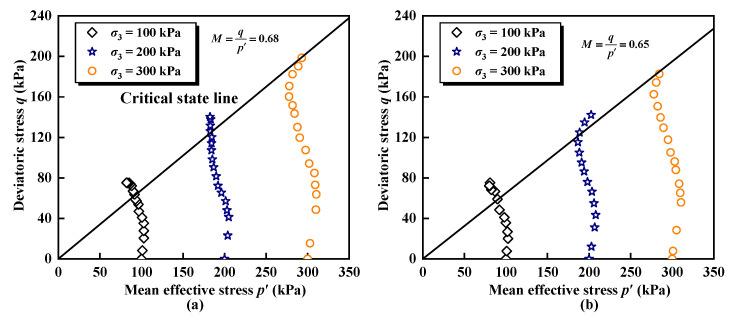
Effective stress paths of ESR: (**a**) Number of freeze–thaw cycles *n* = 0; (**b**) Number of freeze–thaw cycles *n* = 15.

The mechanical behavior of the soil can be elucidated using the critical state framework. The ESR’s effective stress path (ESP) and critical state line (CSL) during various freeze–thaw cycles are illustrated on the *p*′-*q* plane, depicted in Figure 3.

Where *p*′ is the mean effective stress, *p*′ = (*σ*_1_ + 2*σ*_3_)/3 − Δ*u*, and Δ*u* is the pore water pressure.

At the beginning of loading, the slope of the effective stress path is steeper and closely aligns with the total stress path due to the lower pore water stress pressure. Tang et al. [55] suggest that this behavior may be related to the characteristics of the soil after saturation. As strain increases, the pore water pressure gradually increases, causing the effective stress path to shift to the left, indicating that the sample tends to shrink during shear [56]. Under a given condition of freeze–thaw cycles, the slope of the effective stress path for the ESR decreases as confining pressure increases. This decline may be attributed to the rapid increase in particle fragmentation at higher confining pressure, along with variations in relative density, particle size, and particle angularity during shear [57].

**Figure 4:**
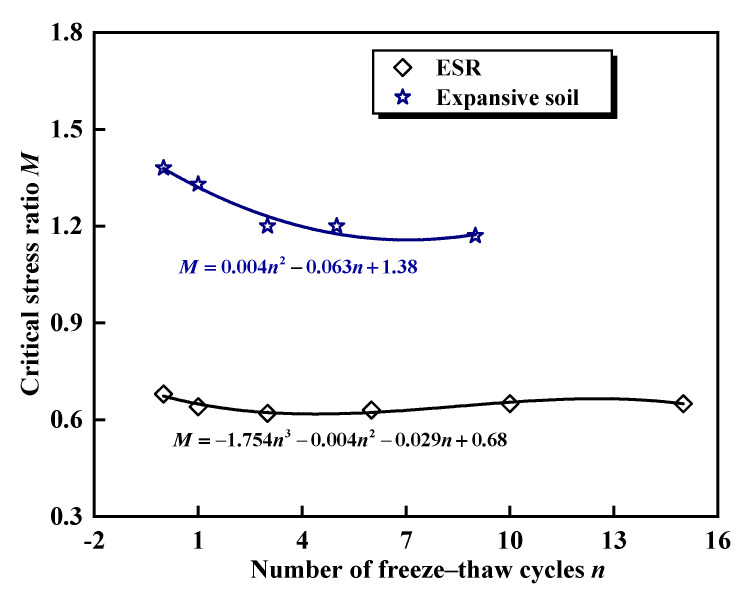
Variation of critical stress ratio under different freeze–thaw cycles, using Tang et al.’ findings as comparison [55].

From Equation (36), the slope *M* of the straight line is obtained by fitting each critical state point with a line that passes through the origin, where *M* represents the critical state ratio. Figure 4 illustrates the variation of the critical stress ratio under different freeze–thaw cycles. It is evident that *M* initially decreases and then gradually stabilizes as the number of freeze–thaw cycles increases. When the number of freeze–thaw cycles is three, the maximum reduction amplitude is 8.8%. With the increase in the number of freeze–thaw cycles, the interface bond strength between the rubber fibers and expansive soil decreases, and the accumulation of plastic deformation leads to a reduction in the critical stress ratio. Tang et al. [55] observed similar patterns while investigating the effects of freeze–thaw cycles on the mechanical properties of expansive soil, attributing these patterns to repeated phase changes in pore water that lead to the formation and expansion of cracks. Niu et al. [58] concluded that freeze–thaw cycles transform some structural units of the sample into a frictional band, resulting in the strength of the freeze–thaw sample being lower than that of the unfrozen sample. In comparison to the findings of Tang et al. [55], the amplitude of reduction in the critical stress ratio of the ESR was less pronounced. This is due to the fact that rubber fibers enhance the tensile strength and toughness of the soil, and the interface bonding between them and the expansive soil matrix plays a critical role in resisting cracking and delamination of the soil mass. From a micromechanical perspective, rubber fibers can absorb and disperse external loads, reducing local stress concentrations and lowering the risk of internal microcrack propagation.

(36) $$M=\frac{q}{p\prime }$$

#### 4.1.2. Effect of Freeze–Thaw Cycles on Elastic Modulus

**Figure 5:**
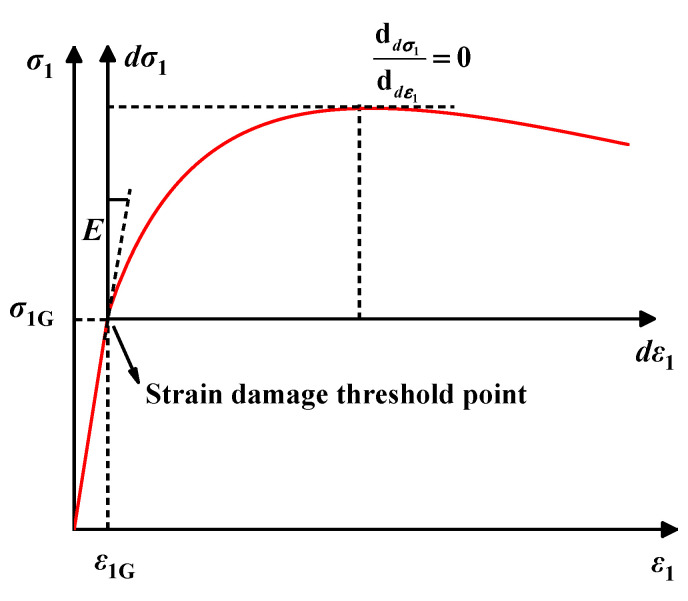
Stress–strain curve of ESR.

The elastic modulus is the key parameter that characterizes soil deformation and stability. Many researchers have defined the elastic modulus as the ratio of the increment of deviatoric stress to the increment of axial strain at 1% axial strain [59]. However, the 1% strain criterion may not provide an accurate representation. Therefore, in this paper, we propose using the ratio of the increment of deviatoric stress at the strain damage threshold to the increment of axial strain at the same threshold as the elastic modulus of the ESR, as illustrated in Figure 5. This relationship can be expressed as Equation (37).

(37) $$E=\frac{\Delta \sigma }{\Delta \varepsilon }=\frac{\sigma_{1G}-\sigma_{0}}{\varepsilon_{1G}-\varepsilon_{0}}$$

where Δ*σ* is the increment of deviatoric stress; Δ*ε* is the increment of axial strain; *σ*_1G_ is the deviatoric stress corresponding to an axial strain of *ε*_1G_; and *σ*_0_ and *ε*_0_ are the initial stress and strain, respectively.

**Figure 6:**
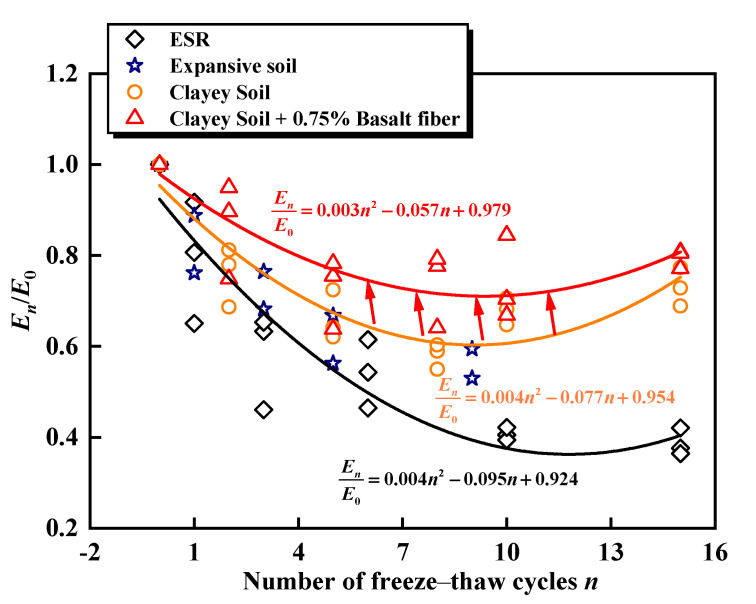
Variation of normalized elastic modulus under different freeze–thaw cycles [55,61].

The elastic modulus is normalized by dividing the elastic modulus of the ESR subjected to a specific number of freeze–thaw cycles by the elastic modulus without freeze–thaw cycles. This approach yields the normalized elastic modulus for different numbers of freeze–thaw cycles, as illustrated in Figure 6. The figure demonstrates a decrease in the elastic modulus for both unreinforced and reinforced samples after undergoing freeze–thaw cycles. For fewer than ten freeze–thaw cycles, the *E*_n_/*E*_0_ ratio decreases significantly; however, when the number of cycles exceeds ten, this ratio stabilizes [60]. When the number of freeze–thaw cycles reaches fifteen, the normalized elastic modulus of ESR decreases to 42.1%. Kravchenko et al. [61] investigated the effects of freeze–thaw cycles on the mechanical properties of both basalt fiber-reinforced clay and unreinforced soil. They found that the fibers effectively mitigated the reduction in the elastic modulus caused by freeze–thaw cycles. Notably, at eight freeze–thaw cycles, the effect was most pronounced, with *E*_n_/*E*_0_ increasing from 0.594 to 0.715, representing a 20.4% increase.

### 4.2. ESR Damage Characterization Analysis

**Figure 7:**
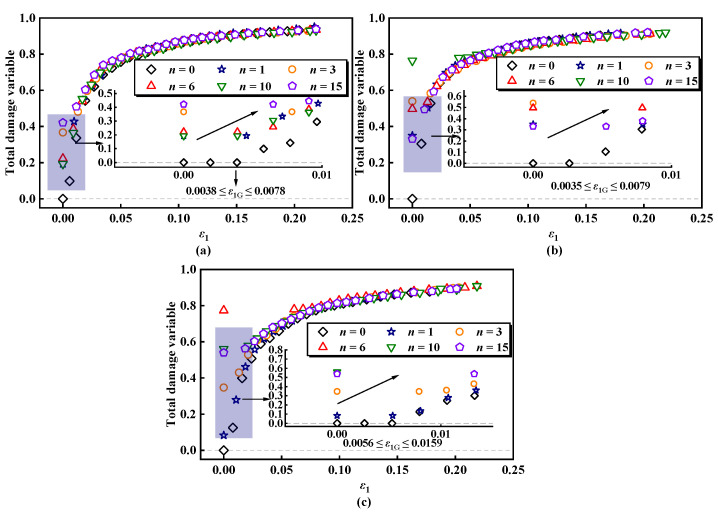
Evolution curves of ESR damage under different confining pressures: (**a**) *σ*_3_ = 100 kPa; (**b**) *σ*_3_ = 200 kPa; (**c**) *σ*_3_ = 300 kPa.

**Figure 8:**
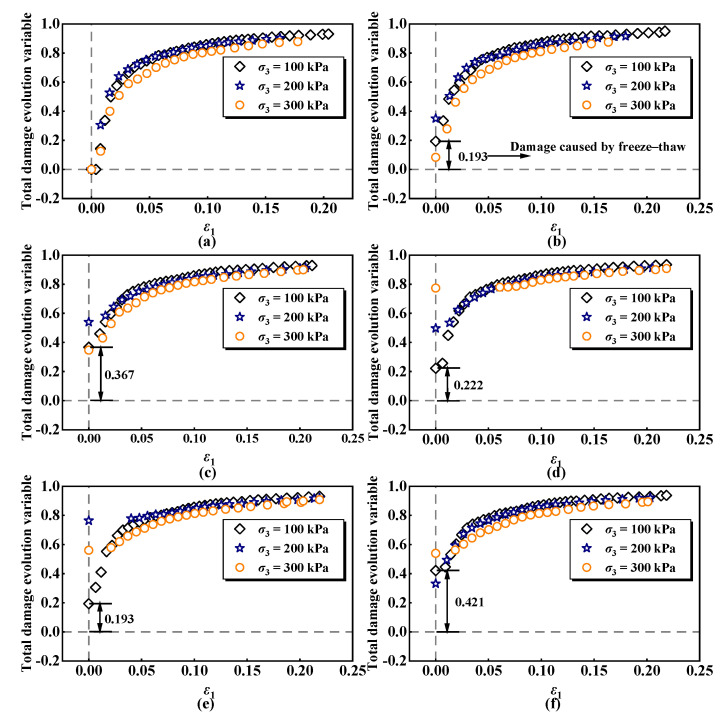
Evolution curves of ESR damage under different freeze–thaw cycles: (**a**) *n* = 0; (**b**) *n* = 1; (**c**) *n* = 3; (**d**) *n* = 6; (**e**) *n* = 10; (**f**) *n* = 15.

The damage evolution curves of ESR under freeze–thaw loading are derived from the calculations. Figure 7 displays the total damage evolution curves of ESR at a specific confining pressure for various numbers of freeze–thaw cycles, while Figure 8 presents the total damage evolution curves of the ESR under different confining pressures for a fixed number of freeze–thaw cycles. It can effectively illustrate the three stages of compression deformation in the ESR:

(1) Weak damage stage: The strain does not reach the strain damage threshold (*ε*_1_ ≤ *ε*_1G_). During this stage, internal microcracks and micropores gradually close under compression. As the axial load increases, these microdefects are further compacted and closed, resulting in a linear elastic stress–strain response. At this stage, the damage to the sample is solely due to the freeze–thaw cycle, and no new damage is generated.

(2) Damage development stage: The strain reaches the damage threshold (*ε*_1_ ≥ *ε*_1G_). As the strain continues to increase, the ESR begins to yield and enters the plastic deformation phase. During this time, damage to the ESR evolves and expands rapidly, with the initiation and development of microcracks.

(3) Failure stage: The microcracks continue to develop, merge, and penetrate to form macrocracks, ultimately converging into the main failure surface. As a result, the damage approaches a value of 1.

As shown in Figure 7, it is evident that the total damage deterioration of ESR is positively correlated with the number of freeze–thaw cycles. During the freezing process, the liquid water in the sample converts to solid ice. Given the high water content of the saturated ESR, the growth of ice crystals causes the ESR to contract, but this contraction is insufficient to offset the displacement caused by the expansion during freezing. As the freeze–thaw cycle progresses, the ESR undergoes repeated freezing and thawing, resulting in the gradual expansion of cracks and consequent damage to the ESR. Macroscopic manifestations include a reduction in ESR stiffness and strength. Zhang et al. [62] explored the damage characteristics of red sandstone under freeze–thaw cycles and load, discovering similar phenomena. The strain damage threshold *ε*_1G_ of the ESR is positively correlated with the number of freeze–thaw cycles. When *σ*_3_ = 100 kPa, the strain damage threshold *ε*_1G_ increases from 0.0038 to 0.0078 as the number of freeze–thaw cycles increases from zero to fifteen. As shear occurs, the damage evolution curves for different numbers of freeze–thaw cycle counts gradually intersect. This phenomenon occurs because the rubber fibers act as tensile elements between soil particles, resulting in the ESR displaying evident plastic behavior.

As shown in Figure 8 the damage of ESR gradually decreases with the increase in confining pressure for a given number of freeze–thaw cycles, indicating that confining pressure can significantly influence the damage deterioration process of ESR. This effect occurs because a higher confining pressure induces a rearrangement of soil particles, which helps compact or close the cracks and fissures formed during the freeze–thaw cycles. Consequently, ESR demonstrates improved damage resistance at higher confining pressures [60]. Moreover, as the number of freeze–thaw cycles increases, the initial damage caused by these cycles also rises. Specifically, the initial damage of ESR increased by 0.42 when the number of freeze–thaw cycles was raised from zero to fifteen at a confining pressure of 100 kPa. However, the initial damage value decreased at seven and nine cycles, which may be attributed to the self-repair effect of the material under specific cycles, microstructural changes caused by moisture absorption and evaporation, stress release phenomena.

The total damage of ESR is influenced by confining pressure, strain, and the number of freeze–thaw cycles, with these three factors interacting with one another in their impact on the mechanical behavior in ESR. The equation for the rate of total damage evolution can be derived based on the work of Zhang et al. [62]:

(38) $${\dot{D}}_{m}=\left(1-D_{n}\right)\frac{\partial D}{\partial \varepsilon_{1}}+\left(1-D_{n}\right)\frac{\partial D}{\partial \sigma_{3}}+\left(1-D_{n}\right)\frac{\partial D}{\partial n}$$

**Figure 9:**
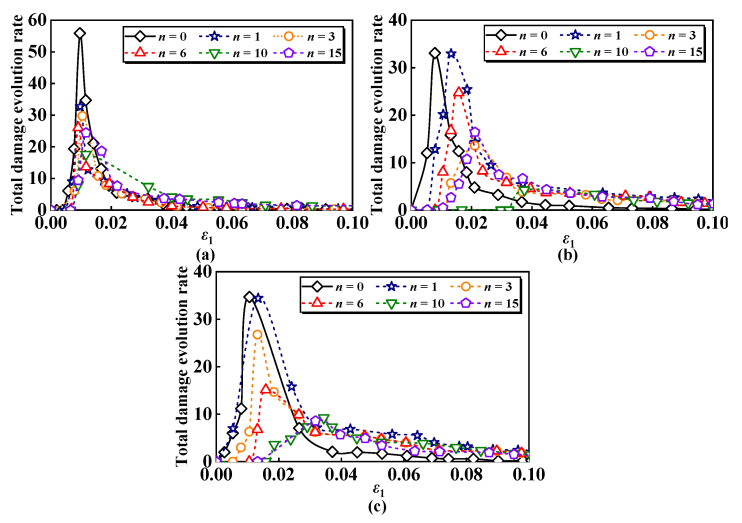
Evolution curves of damage ratio of ESR under different confining pressures: (**a**) *σ*_3_ = 100 kPa; (**b**) *σ*_3_ = 200 kPa; (**c**) *σ*_3_ = 300 kPa.

**Figure 10:**
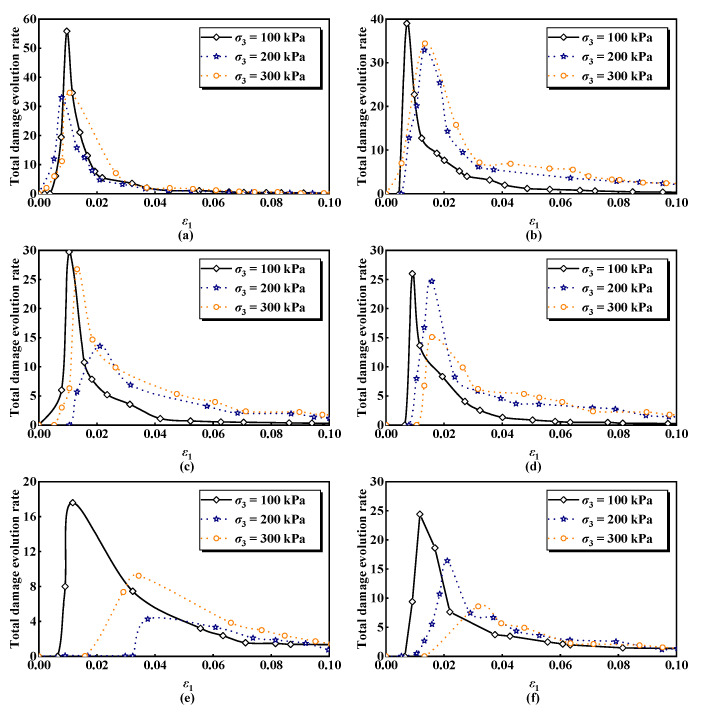
Evolution curves of damage ratio of ESR under different freeze–thaw cycles: (**a**) *n* = 0; (**b**) *n* = 1; (**c**) *n* = 3; (**d**)*n* = 6; (**e**) *n* = 10; (**f**) *n* = 15.

Figure 9 and Figure 10 illustrate the evolution curves of the total damage rate of ESR, calculated using Equation (38). The area enclosed by these damage evolution rate curves represents the damage variable. Figure 9 presents the curve of the total damage evolution rate of ESR across different cycle counts for a specific confining pressure, while Figure 10 depicts the curve at various confining pressures for a set number of freeze–thaw cycles. The damage evolution rate under different confining pressures and freeze–thaw cycles exhibits a consistently upward-convex trend. Similarly, the damage evolution rate curves effectively reflect the three stages of compressive deformation in ESR:

(1) Weak damage stage: The strain does not exceed the strain damage threshold (*ε*_1_ ≤ *ε*_1G_). The total damage evolution rate remains at 0 and unchanged, as internal microdefects gradually closed without the introduction of new damage.

(2) Damage development stage: Once the strain reaches the strain damage threshold (*ε*_1_ ≥ *ε*_1G_), micropores and microcracks begin to increase and expand. This results in an increase in ESR porosity, leading to the onset of yielding and a transition into the plastic deformation stage. Consequently, the damage evolution rate rises rapidly, ultimately reaching its peak.

(3) Failure stage: At this point, the pores expand to form fracture surfaces, causing structure damage to the ESR. As a result, the damage evolution rate gradually approaches 0.

Figure 9 shows that under specific confining pressure, the peak total damage evolution rate of ESR gradually decreases as the number of freeze–thaw cycles increases. Notably, the peak total damage evolution rate declines from 55.8 to 24.4, representing a deduction of 56.3%, when the number of freeze–thaw cycles increases from zero to fifteen cycles at a confining pressure of 100 kPa. After reaching this number of freeze–thaw cycles, the strain of the total damage evolution rate increases at the peak, a trend that is more pronounced at a higher confining pressure. This indicates an enhancement in the plasticity of the ESR.

Figure 10 illustrates that, for a given number of freeze–thaw cycles, the peak of the total damage evolution rate of the ESR gradually decreases with an increase in confining pressure, while the corresponding axial strain at the peak increases, indicating pronounced plasticity. When *n* = 15 cycles, the peak total damage evolution rate decreases from 24.4 to 8.6, representing a reduction of 64.8% as the confining pressure rises from 100 kPa to 300 kPa. This demonstrates that increasing confining pressure inhibits the evolution rate of ESR damage, leading to a gradual transition toward plastic failure.

### 4.3. Model Validation

**Figure 11:**
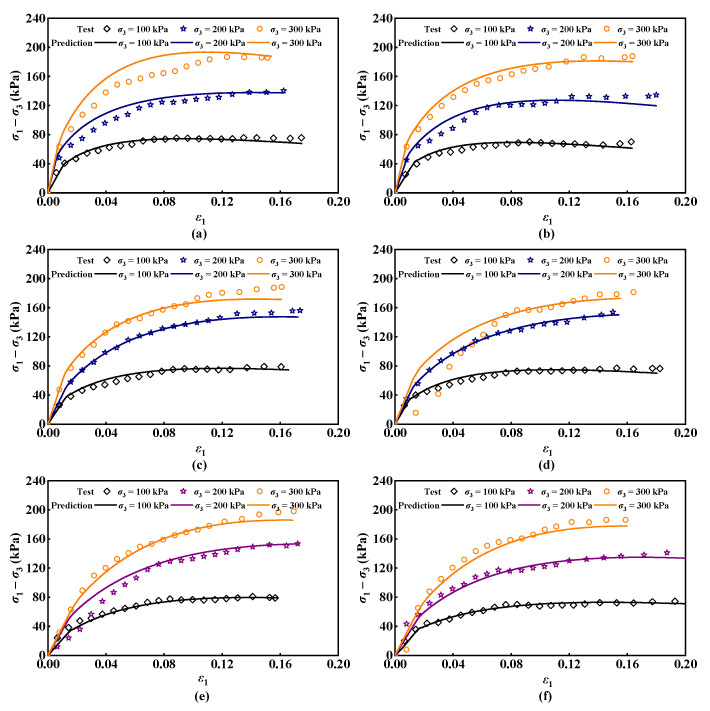
Comparison of theoretical and experimental curves for the ESR damage model: (**a**) *n* = 0; (**b**) *n* = 1; (**c**) *n* = 3; (**d**) *n* = 6; (**e**) *n* = 10; (**f**) *n* = 15.

The parameters *m* and *F*_0_ are fitted as presented in Table 4. Once these parameters are established, the stress of ESR under various confining pressures and freeze–thaw cycles can be calculated using Equation (27) and compared with the experimental data, as illustrated in Figure 11. It is evident that the theoretical curve of the ESR damage constitutive model developed in this paper aligns well with the experimental data. The fitting degree is highest under the 100 kPa condition, indicating that the ESR damage constitutive model can accurately reflect the mechanical behavior of ESR under different confining pressures and freeze–thaw cycle conditions. Moreover, as the number of freeze–thaw cycles increases, the fitting degree of the model improves. Under a confining pressure of 100 kPa, when the number of freeze–thaw cycles is zero, the peak value of the stress in the damage model differs from the experimental data by 9.8%; when the number of freeze–thaw cycles is fifteen, the peak value of the stress in the damage model differs from the experimental data by 3.2%. This is because with an increasing number of freeze–thaw cycles, the development of fissures in the soil becomes more uniform and abundant, allowing the damage variable to be better represented, which in turn enhances the model’s fitting degree. Conversely, under high confining pressure conditions, some internal fissures in the soil close, resulting in an increase in soil strength and a significant rise in the stress damage threshold of the soil. This affects the results simulated by the model, leading to a decrease in the fitting degree.

**Figure 12:**
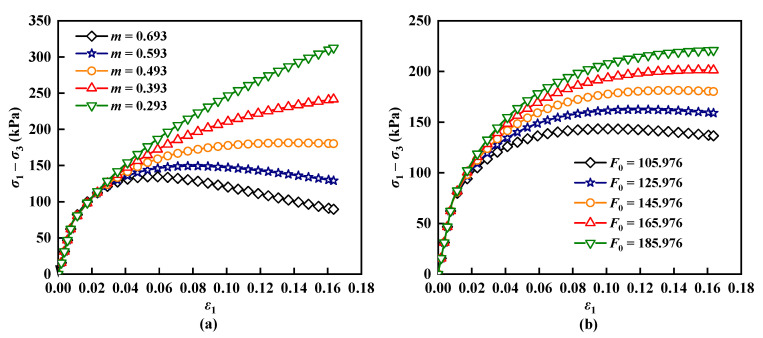
Effect of parameters *m* and *F*_0_ on prediction results: (**a**) *m*; (**b**) *F*_0_.

In this study, a sensitivity analysis of parameters *m* and *F*_0_ was conducted and the effects of these parameters on the prediction results are illustrated in Figure 12. As shown in Figure 12a, parameter *m* primarily influences the peak stress and the degree of strain softening; as *m* increases, the peak stress decreases while strain softening becomes more pronounced. In Figure 12b, it is evident that parameter *F*_0_ mainly affects both the peak stress and peak strain; as *F*_0_ increases, both the peak stress and peak strain rise.

## 5. Conclusions

To investigate the effects of freeze–thaw cycles and confining pressure on the mechanical properties of ESR, consolidation and undrained triaxial tests were conducted under various freeze–thaw conditions. The changes in ESR effective stress paths, critical state line, and the elastic modulus during freeze–thaw cycles were illustrated using the critical state framework. Additionally, based on the theory of continuous damage mechanics, a damage constitutive model for ESR that incorporates confining pressure and freeze–thaw cycles was developed. The main conclusions are as follows:

(1) The effective paths of ESR under different freeze–thaw cycle counts are similar. Under the influence of freeze–thaw cycles, both the elastic modulus and stress ratio decrease; however, the interaction between rubber fibers and soil particles mitigates the reduction in elastic modulus.

(2) In the initial stage, when the strain has not reached the damage threshold, microcracks close, and the damage is provided by the freeze–thaw cycles. Once the threshold is reached, the material begins to yield, and damage evolves rapidly, forming a main failure plane, with the damage value approaching 1.

(3) Freeze–thaw cycles lead to an initial damage value for ESR before shear, which increases with the number of cycles. Under the same confining pressure, the total damage decreases as the number of cycles increases, indicating that the rubber fibers enhance plastic behavior.

(4) Confining pressure limits the development of microcracks, and damage decreases as confining pressure increases, strengthening the ESR. Under the same strain, the total damage value decreases with increasing confining pressure.

(5) A damage constitutive model for ESR considering confining pressure and freeze–thaw cycles has been established. When the number of freeze–thaw cycles is 15 and the confining pressure is 100 kPa, the peak value of the stress in the damage model differs from the experimental data by a small margin of 3.2%.

This study emphasizes the potential application of rubber fibers in backfilling in frozen soil areas, discussing the effects of freeze–thaw cycles and confining pressure on ESR fracturing and damage, providing a reference for future related research and engineering practices.

## Figures and Tables

**Table 1:** Physical properties of expansive soil.

Properties	Value
Natural moisture content	6.10%
Optimum moisture content	16.00%
Maximum dry density	1.853 g·cm^−3^
Specific gravity	2.73
Plastic limit	22.30%
Liquid limit	57.80%
Plasticity index	35.50
Free swell ratio	71.00%
Quartz	58.6%
Calcite	12.3%
Albite	16%
Montmorillonite	13.1%

**Table 2:** Parameters of samples for ESR testing.

Sample Numbers	*f* (%)	Freeze–Thaw Cycles *n*	Confining Pressure *σ*_3_ (kPa)
ESR1, ESR2, ESR3	0	0	100, 200, 300
ESR4, ESR5, ESR6	5	0	100, 200, 300
ESR7, ESR8, ESR9	10	0	100, 200, 300
ESR10, ESR11, ESR12	15	0	100, 200, 300
ESR13, ESR14, ESR15	20	0	100, 200, 300
ESR16, ESR17, ESR18	10	1	100, 200, 300
ESR19, ESR20, ESR21	10	3	100, 200, 300
ESR22, ESR23, ESR24	10	6	100, 200, 300
ESR25, ESR26, ESR27	10	10	100, 200, 300
ESR28, ESR29, ESR30	10	15	100, 200, 300

**Table 3:** Linear relationship coefficients of $I_{1}∼\sqrt{J_{2}}$.

Fiber Content *f* (%)	*s*	*t*	*R* ^2^
0	0.0350	13.761	0.9849
5	0.0300	12.169	0.9937
10	0.0334	9.246	0.9950
15	0.0385	4.473	0.9995
20	0.0399	2.586	0.9939

**Table 4:** Parameters of the damage model under the combined effects of freeze–thaw and load.

F-T Cycles	*σ*_3_ = 100 kPa			*σ*_3_ = 200 kPa			*σ*_3_ = 300 kPa		
	*E_n_*/MPa	*m*	*F*_0_	*E_n_*/MPa	*m*	*F*_0_	*E*_n_/MPa	*m*	*F*_0_
0	5.332	0.488	66.691	8.805	0.420	89.740	9.151	0.462	192.595
1	4.303	0.549	62.369	5.728	0.566	121.933	8.393	0.493	145.976
3	3.376	0.551	74.816	4.057	0.626	171.457	5.977	0.565	173.513
6	3.278	0.574	75.489	4.094	0.581	163.982	4.971	0.553	171.341
10	2.164	0.692	93.296	3.477	0.648	176.865	3.860	0.765	233.564
15	2.007	0.638	78.918	3.208	0.662	154.548	3.852	0.753	222.195

## Data Availability

The original contributions presented in the study are included in the article, further inquiries can be directed to the corresponding author.
